# Etiology of acute otitis media and serotype distribution of *Streptococcus pneumoniae* and *Haemophilus influenzae* in Chilean children <5 years of age

**DOI:** 10.1097/MD.0000000000005974

**Published:** 2017-02-10

**Authors:** Andres Rosenblut, Carla Napolitano, Angelica Pereira, Camilo Moreno, Devayani Kolhe, Alejandro Lepetic, Eduardo Ortega-Barria

**Affiliations:** a Unidad de Otorrinolaringología, Hospital Sótero del Rio, Puente Alto, Santiago, Chile; b Merck & Co, Sao Paulo, Brazil; at the time of the study Takeda Pharmaceuticals, Sao Paulo, Brazil; c GSK Pharmaceuticals Ltd, Bangalore, India; d GSK Buenos Aires, Argentina; e GSK Panama, Panamá.

**Keywords:** acute otitis media, Chile, *Haemophilus influenza*, *Streptococcus pneumoniae*

## Abstract

The impact of bacterial conjugate vaccines on acute otitis media (AOM) is affected by several factors including population characteristics, bacterial etiology and vaccine conjugation method, carrier, and coverage. This study estimated the baseline etiology, distribution, and antibiotic susceptibility of bacterial serotypes that causes AOM in children aged <5 years in a public setting in Santiago, Chile.

Children aged ≥3 months and <5 years referred to the physician for treatment of AOM episodes (with an onset of symptoms <72 h) were enrolled between September 2009 and September 2010. Middle ear fluid (MEF) was collected by tympanocentesis or by otorrhea for identification and serotyping of bacteria. Antibacterial susceptibility was tested using E-test (etrack: 112671).

Of 160 children (mean age 27.10 ± 15.83 months) with AOM episodes, 164 MEF samples (1 episode each from 156 children; 2 episodes each from 4 children) were collected. Nearly 30% of AOM episodes occurred in children aged 12 to 23 months. *Streptococcus pneumoniae* (41.7% [58/139]) and *Haemophilus influenzae* (40.3% [56/139]) were predominant among the cultures that showed bacterial growth (85% [139/164]). All *Streptococcus pneumoniae* positive episodes were serotyped, 19F (21%) and 14 (17%) were the predominant serotypes; all *Haemophilus influenzae* strains were nontypeable. *Streptococcus pneumoniae* were resistant to penicillin (5%) and erythromycin (33%); *Haemophilus influenzae* were resistant to ampicillin (14%) and cefuroxime and cefotaxime (2% each).

AOM in Chilean children is predominantly caused by *Streptococcus pneumoniae* and nontypeable *Haemophilus influenzae*. Use of a broad spectrum vaccine against these pathogens might aid the reduction of AOM in Chile.

## 1 Introduction

Acute otitis media (AOM) is one of the most common bacterial infections in children aged between 6 months and 3 years.^[1,2]^ AOM is a major health concern in the pediatric population worldwide,^[3]^ with the annual disease burden of AOM estimated to range from 980,000 to 1,500,000 cases in 2009 in Latin America and the Caribbean.^[4]^ Previous reports have indicated that AOM results in substantial childhood morbidity. Nearly 83% of children having at least 1 episode of AOM by the time they were 3 years old.^[1]^ AOM is also one of the prime reason for antibiotic prescription during childhood^[5–8]^ in both developed and developing nations.^[9,10]^

Etiological studies conducted in various countries in the past indicated that *Streptococcus pneumoniae* (*S pneumoniae*) and *Haemophilus influenzae* (*H influenzae*) were the predominant bacterial pathogens found in the middle ear fluid (MEF) samples of AOM cases.^[11–14]^ Other bacterial pathogens responsible for AOM included *Moraxella catarrhalis* (*M catarrhalis*), *Streptococcus pyogenes* (*S pyogenes*), and *Staphylococcus aureus* (*S aureus* [frequently isolated in external otitis cases and is cultured from AOM episodes, suggesting potential contamination due to inappropriate sampling]).^[12,13]^

Currently available pneumococcal conjugate vaccines (PCVs) against AOM include a 10-valent pneumococcal *H influenzae* protein D conjugate vaccine (PHiD-CV) (Synflorix, GSK, Wavre, Belgium) and a 13-valent PCV (PCV13) CRM (Prevnar 13/Prevenar 13, Pfizer/Wyeth, Pearl River, NY, USA).

Studies performed using conjugated pneumococcal vaccines showed the efficacy of PCV-7 and PHiD-CV in young children against invasive pneumococcal disease, pneumonia and AOM, and the safety and immunogenicity/effectiveness of PCV-7, PHiD-CV, and PCV-13.^[15–22]^ In Chile, PCV-7 and PCV-13 have been available since 2004 and 2009, respectively. PHiD-CV was approved in 2009 in Chile, and has been included into the Universal Mass Vaccination (UMV) program of Chile since 2011 using a 3 + 1 schedule and 2 + 1 since 2012.^[23]^ PCV-13, which is only available in the private market, started to be used in infants in UMV since 2016 but only in the metropolitan region. As per the recent World Health Organization estimates, the coverage of 3-dose pneumococcal vaccines in Chile was 54% in 2011, which increased to 82% in 2012 and stabilized until 2015 (90%).^[24]^ Although conjugate vaccines have been implemented in various regions, their impact may vary depending on the geographic variability, etiology of AOM, serotype distribution, and vaccination coverage.^[9]^

The resistance of bacterial pathogens responsible for AOM varies depending on the pattern and type of antibiotic used, local prevalence of strains, and rates of vaccination. As a consequence, although the incidence of resistance varies among different regions, it has increased and spread considerably.^[25,26]^

Studies conducted in the past assessed the etiology and serotype distribution of bacterial pathogens causing AOM in various countries^[27–30]^ including Latin American countries such as Chile,^[31]^ Colombia,^[32]^ Mexico,^[33]^ and Venezuela.^[34]^ However, recent epidemiological data in terms of AOM disease burden in Chile are limited.

The present study was conducted to obtain the baseline epidemiological data on the etiology, serotype distribution, and antibacterial resistance of the bacterial pathogens causing AOM prior to the introduction of PCV vaccine in a public clinical setting in Santiago, Chile.

## 2 Methods

### 2.1 Subjects and study conduct

The present prospective epidemiological study was conducted in Hospital Sótero del Río—Servicio de Otorrinolaringología, in Santiago, Chile, between September 2009 and September 2010.

The study was approved by the investigational center Institutional Review Board and was conducted as per the principles of good clinical practice, local regulations in Chile, and the Declaration of Helsinki. Written informed consent was obtained from the parents/guardians of participating children prior to the start of the study.

Children aged ≥3 months and <5 years, referred to the pediatric emergency room/ear, nose, and throat (ENT) specialist in the study hospital for treatment of AOM episodes were enrolled, consistently with other AOM studies.^[35–37]^ The criteria for enrolment included the onset of symptoms of AOM (<72 h) with one of the functional signs of otalgia (or irritability), conjunctivitis, fever (higher than 37.5°C), and Paradise's criteria (bulging, diffused or localized inflamed tympanic membranes), or spontaneous otorrhea of <24 h. All AOM cases were confirmed using pneumatic otoscopy by ENT specialists. Children were not included in the study if they were hospitalized during diagnosis or treatment of AOM; had otitis externa or otitis media with effusion (i.e., not AOM); had presence of transtympanic aerator; received antibiotics 72 h prior to enrolment as therapy for other illnesses; or received antibiotics prior to the MEF sample collection.

Enrolled children were categorized into 2 groups as recommended for etiology studies on AOM^[38,39]^: children with new AOM episodes with onset <72 h and who did not receive antibiotic therapy (untreated group); and children diagnosed with AOM 48 to 72 h prior to enrollment who received antibiotic therapy but remained symptomatic at the time of enrollment (treatment failure group).

Initial diagnosis of AOM was performed by the pediatrician, where clinical examination was performed (recording of body temperature, otalgia [if present], presence of conjunctivitis, existence of otorrhea (ear discharge) of <24 h, irritability, and digestive problems) followed by examination of tympanic membrane. For suspected AOM cases, an ENT specialist reassessed cases and collected an MEF sample either by performing tympanocentesis or by careful sampling of spontaneous otorrhea (removal and cleaning of the ear canal material and deep aspiration of the MEF material via needle insertion). The latter entailed removing and cleaning the ear canal by deep aspiration of the MEF material through the perforation to minimize contamination and spurious results.

Collected MEF samples were kept at room temperature and transported to designated GSK Vaccines laboratories for bacteriological analysis within an hour after collection.

Assessment of safety involved detection and recording of serious adverse events (SAEs) that may occur during the MEF sample collection procedure.

### 2.2 Laboratory assays

The MEF samples collected from enrolled children reporting AOM episodes were used to culture bacteria on chocolate and blood agar to identify *S pneumoniae*, *H influenzae*, *M catarrhalis*, and *S pyogenes*. Bacterial identification for *S pneumoniae* included optochin test and latex test. *Haemophilus influenzae* were identified by Gram staining; growth on chocolate agar, failure to grow on trypticase agar with added sheep blood, and nutritional requirement of both hemin (Factor X) and nicotine adenine dinucleotide (Factor V). Identification of *S Pyogenes* was based on the presence of β-hemolysis, susceptibility to bacitracin, and positive coagglutination and *M catarrhalis* using Gram staining, where positive cultures showed oxidase reaction and characteristic biochemical profiling. If multiple pathogens were identified, all the identified pathogens were recorded separately.

From *S pneumoniae* and *H influenzae* positive cultures, conventional serotyping was performed using Quellung reaction for *S pneumoniae* and monovalent antisera a, b, c, d, e, f test for *H influenzae*. The *H influenzae* identification was achieved by API method (bioMérieux, Marcy l’Etoile, France) while *Haemophilus haemolyticus* strain was identified by VITEK method (bioMérieux), due to absence of API method.

Antibacterial susceptibility was performed against amoxicillin, cefotaxime, erythromycin, and chloramphenicol using E-tests for *S pneumoniae*, *H influenza*, and *M catarrhalis*. Additional E-tests were performed against penicillin for *S pneumoniae*, and against ampicillin and cefuroxime and a beta-lactamase test (nitrocefin) for *H influenzae* and *M catarrhalis*. The interpretation of the results was according to the Clinical Laboratory Standards Institute.^[31]^

### 2.3 Statistical analyses

Serotype distribution of *S pneumoniae* and *H influenzae* was assessed and the proportions of *S pneumoniae* and *H influenzae* serotypes were calculated with their exact 95% confidence interval, calculated using SAS version 9.22 for Windows.

The primary endpoint was to isolate *H influenzae*, *S pneumoniae*, *M catarrhalis*, and *S pyogenes* from the collected MEF samples.

Total enrolled cohort analysis included all children who were enrolled in the study and from whom informed consent was taken prior to the study. The final analysis included all children who met the inclusion criteria, complied with all the study procedures, with no elimination criteria during the study and from whom laboratory results of the MEF episodes were available.

Children were followed for 1 week after tympanocentesis for any adverse event that occurred.

## 3 Results

### 3.1 Demography

A total of 160 children reporting AOM episodes were included in the final analysis, from whom 164 samples were collected: 156 children reporting 1 episode each (150 in untreated group and 6 in treatment failure group) and 4 children with 2 episodes each (2 in untreated and treatment failure group each). Of these, 146/164 (89.0%) episodes were collected by tympanocentesis and 18/164 (11.0%) from otorrhea. The mean age (standard deviation) of children reporting AOM episodes included in the final analysis was 27.10 (±15.83) months (range 4–59 months) (Table 1). Episodes of AOM occurred predominantly (29.9%) in children aged 12 to 23 months, with 48.8% (80/164) of all episodes occurring in children below 23 months of age (Table 2).

**Table 1:**
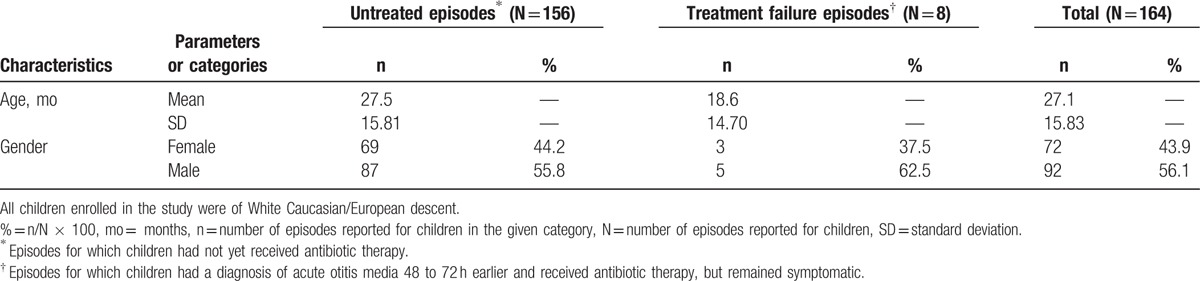
Demographic characteristics (final analysis [N = 164]).

**Table 2:**
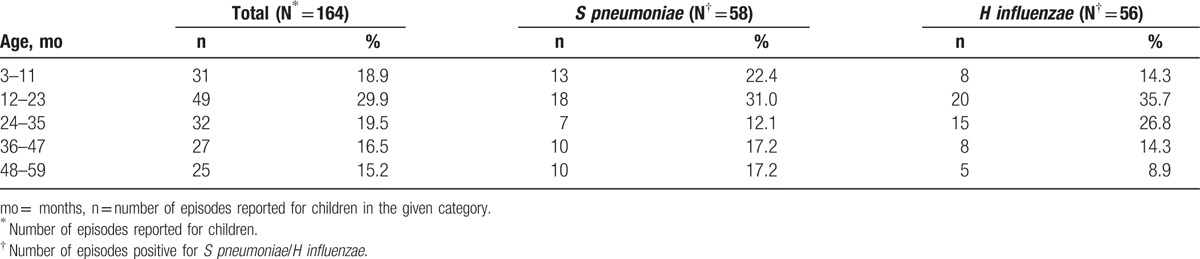
Etiology of acute otitis media episodes by age (final analysis [N = 164]).

### 3.2 Bacterial etiology

Bacterial growth was observed in 84.8% (139/164) of samples that were cultured, which included 12.2% (17/139) and 87.8% (122/139) of the otorrhea and tympanocentesis cases, respectively. Of these, 94.2% (131/139) were positive for at least 1 bacterium (*S pneumoniae*, *H influenzae*, *M catarrhalis*/*Brahanmella catarrhalis* or *S pyogenes*/*Streptococcus* group A) (Table 3). Bulging of tympanic membrane was reported in 64.7% (90/139) of the samples; of which 45.6% (41/90) episodes were positive for *S pneumoniae*.

**Table 3:**
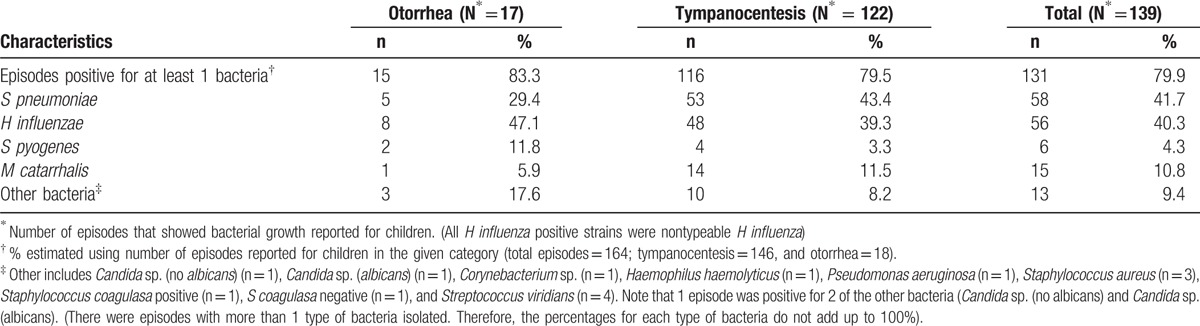
Bacterial etiology of episodes by sample collection mode (final analysis [N = 164]).

*Streptococcus pneumoniae* and *H influenzae* were the predominant bacteria isolated in the episodes that showed bacterial growth, observed in 41.7% (58/139) and 40.3% (56/139), respectively (Table 3). Among the 8 episodes in the treatment failure group, *H influenzae* was identified in 37.5% (3/8) of cases. There was no statistically significant difference in the proportion of *H influenzae* between the untreated (40.5% [53/156]) and treatment failure groups.

Two episodes had cultures positive to both *S pneumoniae* and *H influenzae*. The highest percentage of *S pneumoniae* and *H influenzae* isolates were observed in children aged 12 to 23 months; 31% (18/58) and 35.7% (20/56), respectively (Table 2).

**Figure 1:**
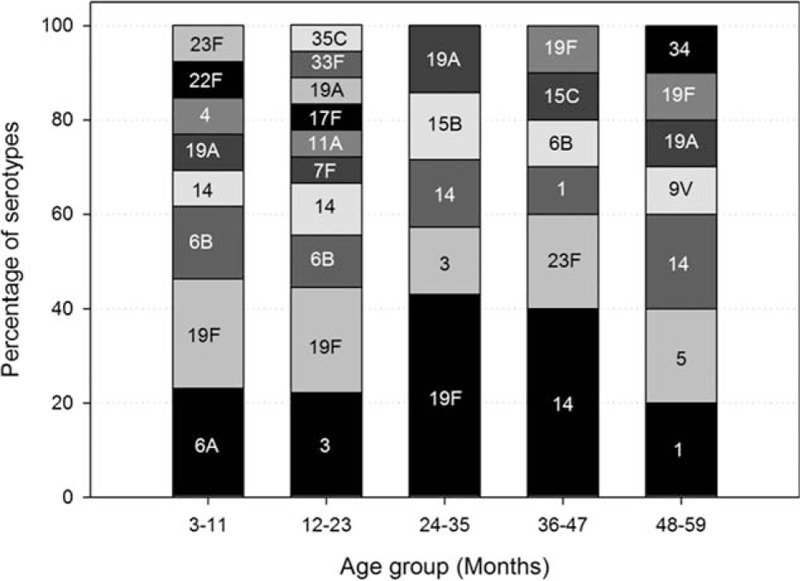
Distribution of *Streptococcus pneumoniae* positive episodes by age and serotype (N = 58).

Among the positive episodes for *S pneumoniae*, the most common serotypes were 19F and 14, observed in 21% (12/58) and 17% (10/58) of episodes, respectively. The other *S pneumoniae* serotypes observed were 3 and 6B (9% each), 19A (7%); 1, 6A, and 23F (5% each); 5 (3%); 4, 7F, and 9V (2% each), with non-PCV types (11A, 15B, 15C, 17F, 22F, 33F, 34, and 35C) accounting for 14% of episodes positive for *S pneumoniae*. The distribution of *S pneumoniae* positive episodes by age is depicted in Fig. 1. All *H influenzae* positive episodes were nontypeable (NTHi).

The occurrence of *S pneumoniae* and *H influenzae* isolates were higher in males (65.5% [38/58]) and (55.4% [31/56]) among cultures that showed bacterial growth, respectively.

Of the 164 episodes obtained from 160 children, in 107 episodes (65.2% [107/164]) children were vaccinated against *H influenza* (at least 1 dose). On the other hand, only 6 episodes (3.7% [6/164]) children were vaccinated with PCV-7 (3 received all 3 doses, 1 received 2 doses, and 2 received 1 dose each). Among the 6 AOM episodes reported by vaccinated children 4 were positive for bacteria assessed: 1 was positive for *S pneumoniae* (serotype 3), 2 were positive for NTHi, and 1 was positive for *M catarrhalis*. Among pneumococcal-unvaccinated children, 85.4% of the episodes cultured bacterial growth (135/158). Among them 42.2% (57/135) and 40.0% (54/135) had episodes positive for *S pneumoniae* and *H influenzae*, respectively.

### 3.3 Seasonal distribution of AOM episodes

**Figure 2:**
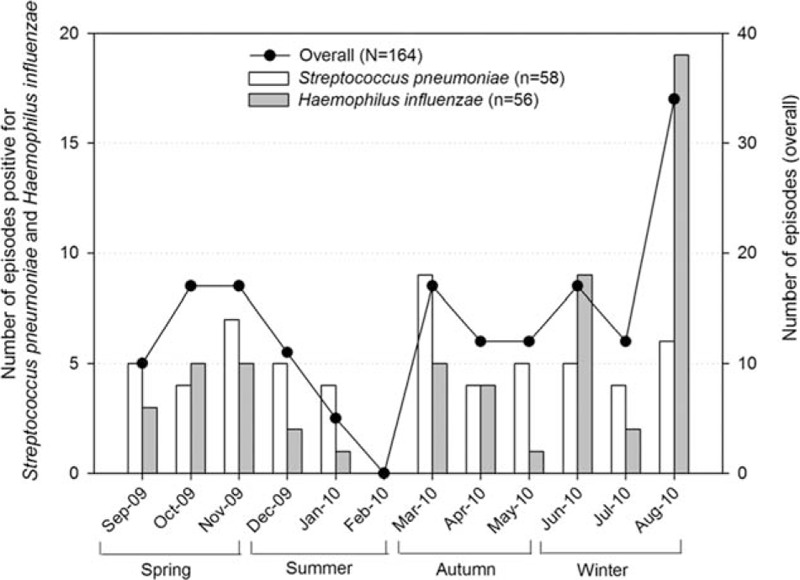
Seasonal distribution of acute otitis media, *Streptococcus pneumoniae* positive and *Haemophilus influenzae* positive episodes included in the final analysis (N = 164).

Although the number of AOM episodes remained fairly even throughout the year, the number of episodes peaked between March 2010 and August 2010, corresponding to autumn and winter seasons in the Southern Hemisphere where Chile is located. The highest number of AOM episodes was observed in August 2010 (34/164), *S pneumoniae* positive episodes in March 2010 (n = 9) and *H influenzae* positive episodes in August 2010 (n = 19) (Fig. 2).

### 3.4 Antibiotic/antibacterial susceptibility

Among the 58 episodes positive for *S pneumoniae*, prevalence of sensitive, intermediate, and resistant strains for penicillin were 52% (30/58), 43% (25/58), and 5% (3/58), respectively. Antibacterial resistance was also observed against erythromycin (33% [19/58]). Serotype 14 was resistant to penicillin (20% [2/10]) and erythromycin (40% [4/10]); serotype 23F was resistant to penicillin (33% [1/3]) and erythromycin (67% [2/3]); serotypes 6A, 6B, and 19F were resistant to erythromycin (67% [2/3], 100% [5/5], and 50% [6/12], respectively).

NTHi strains were resistant to ampicillin (14% [8/56]), cefuroxime (2% [1/56]), and cefotaxime (2% [1/56]). Further, nitrocefin (beta-lactamase test) resulted negative for 88% (49/56) and positive for 12% (7/56) of episodes.

### 3.5 Safety assessment

None of the enrolled children reported any SAEs related to tympanocentesis during the entire study period.

## 4 Discussion

The present study conducted in Chilean children describes the etiology, serotype distribution, and antibacterial resistance of bacterial pathogens involved in AOM. The percentage of bacterial isolation (84.8%) in this study was higher when compared to other studies from Colombia (63%), Mexico (64%), and Venezuela (69%). This could probably be due to use of tympanocentesis for diagnosis of AOM. The results from the present study indicated that the majority of AOM episodes were due to *S pneumoniae* and NTHi, among those with bacterial growth. These results were comparable with that of an etiological study previously conducted in Chile (*S pneumoniae* [37%] and *H influenzae* [24%])^[31]^ and other Latin American countries such as Colombia,^[32]^ Mexico,^[33]^ Venezuela,^[34]^ Costa Rica,^[40,41]^ and Argentina.^[42]^ Furthermore, the present study indicated that *M catarrhalis* and *S pyogenes* were less frequently reported in AOM episodes. These observations reinforce the results from previous studies that assessed the clinical characteristics of *M catarrhalis* and *S pyogenes*.^[43,44]^ In addition, AOM episodes mainly occurred in children aged between 12 and 23 months which is consistent with previous reports.^[2,5]^

Globally, serotypes 3, 6A, 6B, 9V, 14, 19A, 19F, and 23F have been shown to be the most prevalent serotypes of *S pneumoniae* causing AOM. Our present study showed that serotypes 19F, 14, and 3 constituted nearly half (46.6%) of isolated pneumococcal serotypes. These observations also corroborate the findings of earlier studies in the Latin American region, where 19F and 14 were among the top 3 pneumococcal serotypes.^[22,32,40,41]^

A previously conducted study reported that *S pneumoniae* serotypes 14, 5, 6 (B/A), 1, and 19 (F/A) accounted for nearly two-thirds of invasive diseases in Chilean children aged <5 years.^[45]^ In the present study, these invasive *S pneumoniae* serotypes were observed in over 50% of AOM episodes. The proportion of *S pneumoniae* and *H influenzae* positive episodes appeared to be comparable (at least 40.0%) in unvaccinated children. In our study, no more than 6 children out of 160 (3.7%) were vaccinated with a pneumococcal vaccine. The study is based on an approximation of the children being vaccinated and pneumococcal vaccination used (PCV-10, PVC-13, or PHiD-CV) is unknown regarding each case. However, vaccine uptake was mainly for PCV-7 since the enrolled population was from the public sector where use of the vaccine was highly limited due to its cost and because it was not included in the UMV during the conduct of the study.^[23]^ This study reflects mainly the AOM etiology prior to the use of PCV in the universal mass vaccination program for Chile for the non-PCV vaccinated population.

The previous study on the etiology of AOM^[31]^ in Chile showed a slightly higher proportion of Group A *Streptococcus* and slightly lower *Haemophilus*. It is likely that there may have been changes in the local microbiology that may have led to different results in the present study.

The percentage of *S pneumoniae* and *M catarrhalis* were lower and *H influenzae* and *S pyogenes* were higher among otorrhea samples when compared to tympanocentesis (Table 3). Similar data were showed in a previously published study, except that *H influenzae* is found in lower percentaged among otorrhea patients.^[46]^ There was no difference in the distribution of *S pneumoniae* between otorrhea and tympanocentesis patients.

It has been established that increased antibiotic usage against AOM during early childhood may lead to serious consequences like bacterial pathogens developing antibacterial resistance.^[5–8]^ Recent studies had raised concerns with *S pneumoniae* developing resistance against penicillin and other antibiotics.^[47,48]^ Contrary to this, the results of the present study showed only 5.2% of *S pneumoniae* positive episodes to have complete resistance to penicillin, which is in line with previous studies conducted in Chile, Colombia, Mexico, and Costa Rica.^[21,22,25,26,31–33,40,41]^

A limitation of this study was its conduct in a public clinical setting (Hospital Sótero del Río—Servicio de Otorrinolaringología, in Santiago) in Chile where the number of children enrolled might not represent the entire Chilean population. Furthermore, since incidence data were not collected in study, the scope for future ecological comparisons assessing the PCVs prior to and postintroduction into the UMV is limited with this data.

## 5 Conclusions

Etiological assessment of AOM revealed that *S pneumoniae* and nontypeable *H influenzae* were the leading causative bacterial pathogens of AOM in Chilean children aged ≥3 months and <5 years. Since PHiD-CV was introduced in UMV in 2011,^[22]^ assessment of the vaccine's impact on AOM would be of public health interest. In addition, monitoring of the AOM trends is still needed to assess potential impact of PCV vaccines etiology.

## Acknowledgments

The authors would like to thank the physicians, study nurses, and the parents of children who participated in this study. The authors also thank Camila Jhones (Senior Clinical Research Associate) for coordinating this study, Harshith Bhat and Mark Franco for medical writing, Varshini Sreenivas (GSK), Marjorie Vasquez, Jessica Mattos, Vinicius Costa, and Ingrid Leal (all employees of GSK). The authors also thank Business and Decision Life Sciences platform for editorial assistance and manuscript coordination, on behalf of GSK. Pierre-Paul Prevot provided editing support and Gregory Collet coordinated manuscript development and editorial support.
